# Discrepancy between inter- and intra-subject variability in EEG-based motor imagery brain-computer interface: Evidence from multiple perspectives

**DOI:** 10.3389/fnins.2023.1122661

**Published:** 2023-02-13

**Authors:** Gan Huang, Zhiheng Zhao, Shaorong Zhang, Zhenxing Hu, Jiaming Fan, Meisong Fu, Jiale Chen, Yaqiong Xiao, Jun Wang, Guo Dan

**Affiliations:** ^1^School of Biomedical Engineering, Health Science Center, Shenzhen University, Shenzhen, Guangdong, China; ^2^Guangdong Provincial Key Laboratory of Biomedical Measurements and Ultrasound Imaging, Shenzhen, Guangdong, China; ^3^School of Electronic Information and Automation, Guilin University of Aerospace Technology, Guilin, China; ^4^Shenzhen Institute of Neuroscience, Shenzhen, Guangdong, China; ^5^Shenzhen Qianhai Shekou Free Trade Zone Hospital, Shenzhen, Guangdong, China

**Keywords:** brain-computer interface, inter- and intra-subject variability, electroencephalography (EEG), motor imagery, sensorimotor rhythms (SMR)

## Abstract

**Introduction:**

Inter- and intra-subject variability are caused by the variability of the psychological and neurophysiological factors over time and across subjects. In the application of in Brain-Computer Interfaces (BCI), the existence of inter- and intra-subject variability reduced the generalization ability of machine learning models seriously, which further limited the use of BCI in real life. Although many transfer learning methods can compensate for the inter- and intra-subject variability to some extent, there is still a lack of clear understanding about the change of feature distribution between the cross-subject and cross-session electroencephalography (EEG) signal.

**Methods:**

To investigate this issue, an online platform for motor-imagery BCI decoding has been built in this work. The EEG signal from both the multi-subject (Exp1) and multi-session (Exp2) experiments has been analyzed from multiple perspectives.

**Results:**

Firstly we found that with the similar variability of classification results, the time-frequency response of the EEG signal within-subject in Exp2 is more consistent than cross-subject results in Exp1. Secondly, the standard deviation of the common spatial pattern (CSP) feature has a significant difference between Exp1 and Exp2. Thirdly, for model training, different strategies for the training sample selection should be applied for the cross-subject and cross-session tasks.

**Discussion:**

All these findings have deepened the understanding of inter- and intra-subject variability. They can also guide practice for the new transfer learning methods development in EEG-based BCI. In addition, these results also proved that BCI inefficiency was not caused by the subject’s unable to generate the event-related desynchronization/synchronization (ERD/ERS) signal during the motor imagery.

## 1. Introduction

Inter- and intra-subject variability happened pervasively and elusively across different subjects and within the same subject over time for the electroencephalography (EEG) recording (Saha and Baumert, 2020). The inter-subject variability could be attributed to the factors of age, gender, and living habits, which would be related to the brain topographical and electrophysiology (Seghier et al., 2004; Herzfeld and Shadmehr, 2014; Wu et al., 2014; Seghier and Price, 2018; Antonakakis et al., 2020). The intra-subject variability would be explained as the changes of psychological and physiological, such as fatigue, relaxation, and concentration (Smith et al., 2005; Meyer et al., 2013; Nishimoto et al., 2020; Trinh et al., 2021; Hu et al., 2022).

Inter- and intra-subject variability poses a major challenge in the field of EEG-based brain-computer interfaces (BCIs) (Ray et al., 2015; Saha et al., 2017; Lee et al., 2019; Chikara and Ko, 2020; Wei et al., 2021; Huang et al., 2022). By detecting the event-related desynchronization/synchronization (ERD/ERS) in sensorimotor rhythms (SMR), motor imagery-based BCI (MI-BCI) has been proposed for neuro-rehabilitation applications, ranging from patients with motor disabilities, severe muscular disorders, and paralysis to the restoration of limb movements (Wolpaw and Wolpaw, 2012; Mane et al., 2020). However, a well-trained BCI model from a certain subject could not be directly applied to the other subject. Further, previous studies have shown the BCI inefficiency problem that there would be 10 to 50% of users are unable to operate the MI-BCI systems (Vidaurre and Blankertz, 2010; Liu et al., 2020). Even on the same subject, the performance of the BCI system would degrade over time. The existence of inter- and intra-subject variability leads to the decline of the generalization of conventional machine learning, which in turn limits the application of MI-BCI to practicality (Ahn and Jun, 2015; Saha et al., 2017).

Under the conventional machine learning framework, the training set and testing set need to be independent and identically distributed (I.I.D) (Duda and Hart, 2006). However, the inter- and intra-subject variability make the assumption of the I.I.D condition no longer tenable. By relaxing the I.I.D assumption’s limitation requirements, transfer learning is considered an effective way to improve the model’s reusability and generalization for inter- and intra-subject variability (Jayaram et al., 2016; Pan, 2020). A series of methods have been proposed to transfer knowledge from the source domain to the target domain. Invariant representation aims to find some invariant learning model across the different sessions or subjects, such as regularized common spatial pattern (CSP) and invariant CSP (Blankertz et al., 2007; Cheng et al., 2017; Xu et al., 2019). With the development of deep learning techniques, domain adaptation methods have been proposed and almost exclusively dominated the field of BCI application (Li et al., 2010; Liu et al., 2012; Samek et al., 2013; Fukunaga, 2013; Dagaev et al., 2017; Azab et al., 2019; Hong et al., 2021). Some end-to-end advantages and stronger feature learning ability and has received more and more attention (Autthasan et al., 2021).

Although the challenge of inter- and intra- subject variability to the real application has been gradually noticed and transfer learning can compensate for the performance decrease to a certain extent, the understanding of the inter- and intra- subject variability is still limited. Most researchers treated inter- and intra- subject variability as the similar type of problem (Jayaram et al., 2016). While both inter- and intra- subject variability would lead to a change in the feature distribution, their differences are underexplored. Firstly, how the subject-specific and time-variant ERD/ERS patterns vary during sensorimotor processing on the signal preprocessing level, and whether the two types of variations are similar or not. Secondly, whether the inter- and intra- subject variability would lead to the covariate shifts in different situations on the feature extraction level? Finally, to overcome the change in feature distribution, whether different strategies should be adopted to deal with the inter- and intra- subject variability for training the machine learning model on the classification level?

This work aims to further explore the internal influence of the inter- and intra-subject variability of EEG decoding in the application of MI-BCI and the difference between them. A real-time MI-BCI online decoding platform has been built and both multi-subject and multi-session EEG signals have been recorded on this platform. In the following, the problem of inter- and intra-subject variability has been investigated from multiple perspectives. Firstly, compared with the classification accuracies, we performed the time-frequency analysis to compare the variability in the cross-subject and cross-session cases on the signal level. After that, the feature distribution for the covariate shifts during the cross-subject and cross-session transfer has been compared on the feature extraction level. Finally, the training sample selection strategies have been investigated on the classification level for both the cross-subject and cross-session tasks.

## 2. Materials and methods

### 2.1. Participant

This study included ten healthy subjects (aged 20 to 25 years old, half of whom were females). All subjects reported that they had no history of neurological or psychiatric disorders. None of the subjects had a history of neurological or psychiatric disorders. All subjects gave their written informed consent before the data recording. Ethical approval of this study was approved by Medical Ethics Committee, Health Science Center, Shenzhen University (No. PN-202200029).

### 2.2. Experimental platform

#### 2.2.1. System framework

**FIGURE 1:**
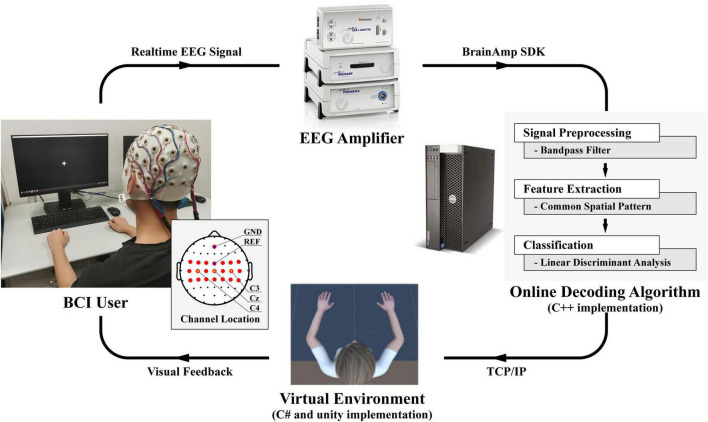
The framework of the online platform of the BCI system.

The framework of the online platform of the BCI system is shown in Figure 1. The real-time EEG signal is first acquired by an EEG amplifier (BrainAmp, Brain Products GmbH, Gilching, Germany). With the BrainAmp SDK, a C++-implemented online decoding algorithm is used for real-time signal preprocessing, feature extraction, and classification. After that, the results would be sent to the virtual reality by Transmission Control Protocol/Internet Protocol (TCP/IP), in which the virtual character would perform left-hand or right-hand grasping actions according to the classification results. The virtual reality is made by Unity software with the C# implemented. Finally, the avatar’s movements are visual feedback to the subjects. So far, we have formed a closed-loop platform for online BCI decoding and rehabilitation training.

#### 2.2.2. Signal preprocessing

The real-time EEG signals were acquired *via* a multichannel EEG electrode system (Easycap) and an EEG Amplifier (BrainAmp, Brain Products GmbH, Germany) using BrainAmp SDK. The sampling rate was 5,000 Hz, and the FCz channel was used as a reference. During the experiment, 20 EEG electrode channels over the motor sensory area surrounding C3, Cz, and C4 were selected for EEG signal recording, which were FC5, FC3, FC1, FC2, FC4, FC6, C5, C3, C1, Cz, C2, C4, C6, CP5, CP3, CP1, CPz, CP2, CP4, CP6 as illustrated in Figure 1. To ensure the quality of EEG recording, the contact impedance between EEG electrodes and cortex was required to be lower than 20 kΩ.

**FIGURE 2:**
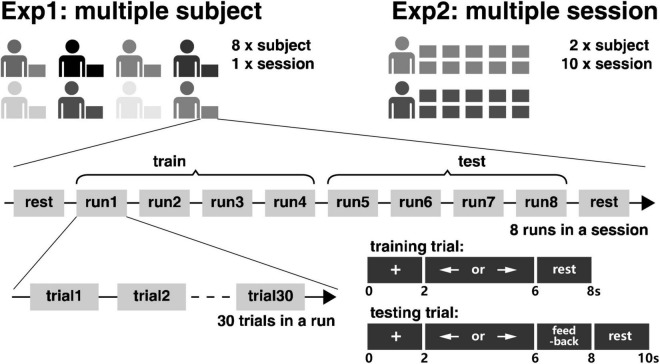
The experimental paradigm.

For signal pre-processing, the acquired signal was firstly down-sampled from 5,000 to 250 Hz, and 50 Hz power frequency interference was removed by a 4-order Butterworth notch filter. After that, the MI EEG signal from 8 to 30 Hz was extracted by a 4-order Butterworth bandpass filter. The time window from 2 to 6 s is selected for single trial data extraction, the motor imagery task starts in the 2nd second and ends in the 6th second, see Figure 2 for details.

#### 2.2.3. Feature extraction

The CSP algorithm was applied for SMR feature extraction (Blankertz et al., 2007; Fukunaga, 2013). Firstly, the normalized spatial covariance matrix was obtained for each trial.


(1) $$\Sigma_{i}=\frac{X_{i}^{T}\,X_{i}}{t\,r\,a\,c\,e\,\left\{X_{i}^{T}\,X_{i}\right\}}$$

in which *X_i_* ∈ *R^C^*^×^*^T^* is the EEG signal of the trial *i*, *C* represents the number of electrode channels, *T* represents the number of samples for each channel. After the training runs, the averaged spatial covariance matrices Σ*^r^* and Σ*^l^* can be obtained for left and right motor imagery trials. The CSP algorithm can be realized by solving the following generalized eigenvalue problem (Blankertz et al., 2007; Lotte et al., 2018).


(2) $$\Sigma^{r}\,w=\lambda \,\Sigma^{l}\,w$$

three pairs of spatial filters *w* corresponding to the largest and smallest eigenvalues λ can be obtained. The spatial filters aim to maximize the variance of one type of EEG signal and simultaneously minimize another type of EEG signal. After that, six-dimensional features can be extracted as the logarithmic band power of the spatially filtered EEG signals, which were used for online classification.

#### 2.2.4. Classification

The classifier Linear discriminant analysis (LDA) (Duda and Hart, 2006) was used for pattern recognition. Let


(3) $$S=\sum_{k}^{2}\sum_{x\in D_{k}}\left(x-\mu_{k}\right)\,{\left(x-\mu_{k}\right)}^{T}$$

to be the within-class scatter matrix, and (μ_1_−μ_2_)(μ_1_−μ_2_)^*T*^ to be the between-class scatter matrix, in which


(4) $$\mu_{k}=\frac{1}{N_{k}}\,\sum_{x\in D_{k}}x$$

is the mean value of the features for class *k*, *k* = 1,2 for the motor imagery tasks of the left hand and right hand. LDA aims to maximize the between-class scatter matrix and minimize the within-class scatter matrix with the linear discrimination function


(5) $$g\,\left(x\right)=w^{T}\,x+b$$

in which *x* represents a single feature vector extracted by CSP,


(6) $$w=S^{-1}\,\left(\mu_{1}-\mu_{2}\right)$$

and


(7) $$b=-\frac{1}{2}\,{\left(\mu_{1}+\mu_{2}\right)}^{T}\,S^{-1}\,\left(\mu_{1}-\mu_{2}\right).$$

### 2.3. Experimental paradigm

The study included two experiments for MI-BCI, which are termed Exp1 and Exp2. As is shown in Figure 2, eight subjects took participated in Exp1 for only one session for the investigation of inter-subject variability, and two subjects took participated in Exp2, which is the same as Exp1. But the two subjects need to come for the experiment for 10 sessions on different days for the investigation of intra-subject variability.

In the experiment, the subjects were asked to sit in front of the screen and complete the corresponding tasks according to the instructions on the screen. At the beginning and end of the experiment, a length of 60 s of resting-state EEG was recorded, in which the subjects were asked to fix their eyes and stare at the cross in the center of the monitor screen.

As illustrated in Figure 2, a total of 240 trials of motor imagery trials were randomly arranged in the 8 runs of the BCI experiment, in which 120 trials were for the left hand and 120 trials were for the right hand. The subject would have a rest between two runs. The first four runs were used for training the online BCI system in the experiment. At the beginning of each trial, a cross appears on the screen for 2 s. The subjects to prepare the following tasks, there would be a left or right arrow appearing randomly on the screen. The subjects need to perform the corresponding left- or right-hand motor imagery for the following 4 s. With the arrow disappearing, the subjects would have a rest between two trials. The last four runs were used for testing the online BCI system. Different from the trials in the training phase, there were 2 s of visual feedback after the 4 s of motor imagery in the testing phase. Hence, the subjects can immediately know whether their motor imagery was correctly recognized by the online BCI system.

### 2.4. Signal-level analysis

The recorded EEG signal was firstly segmented from −2–8 s with a time interval of 0–4 s for motor imagery. Secondly, a wavelet transform was performed for the time-frequency analysis. Baseline correction has been performed by subtraction with the reference interval −2–0 s relative to stimulus onset. By averaging the trials with left and right hands motor imagery, we can have the time-frequency response from 8 to 30 Hz on channels C3 and C4 from each subject and each session.

Suppose *P*_pre_ is the mean value of the EEG power from 8 to 30 Hz and the time interval of −2–0 s and the frequency of 8–30 Hz; *P*_post_ is the mean value of the EEG power from 8 to 30 Hz and the time interval of 0–4 s and the frequency of 8–30 Hz. The event-related desynchronization/synchronization index (ERD/ERS index) (Ma et al., 2022) was calculated on channels C3 and C4 as follows.


(8) $$ERD/ERS\;index=\frac{P_{\text{post }}-P_{\text{pre }}}{P_{\text{pre }}}\times 100\%$$

### 2.5. Feature-level analysis

On the feature level, the EEG signal was used to make the CSP spatial filter and LDA classifier training for each session in Exp1 and Exp2 and then applied to other sessions. For inter-subject analysis (Exp1), the EEG signal from each subject was used for training and testing on all eight subjects. It should be noted that it was also tested on the subject him/herself, which means the training data and testing data are the same in this case. By repeating the analysis eight times on the eight subjects, we could have the 8 8 feature distribution with the corresponding classification accuracies in Exp1. For the intra-subject analysis (Exp2), since both two subjects repeated the experiment 10 times, we would have two 10 10 feature distributions with the corresponding classification accuracies in Exp2.

For data visualization, only the first pair of CSP spatial filters were used for feature extraction and classifier training. Since the aim of the study was to explore the difference between the inter- and intra- subject variability in MI-BCI not to achieve high accuracies, feature space was limited to two-dimension for easy investigation. No advanced methods, like Filter Bank Common Spatial Pattern (FBCSP), with high dimension feature space, have been applied in this work.

For statistical analysis, four statistics have been compared between the feature distribution in Exp1 and Exp2, which are Mean, Std, *Dist*_*w*_ and *Dist*_*b*_. Consider the:


(9) $$x_{c}^{i}=\left[x_{c}^{i\,1},x_{c}^{i\,2}\right]$$

is the two-dimensional feature of the *i*-th trial in class *c*, *i* = 1, 2,…,*n* and *c* = 1 *or* 2 for the class of left and right motor imagery, *n* = 120 in this study.

• **Mean:** the mean value of the two-dimension features from all trials of the EEG signals within a session.


(10) $$\mu =\frac{1}{2\,n}\,\sum_{c=1}^{2}\sum_{i=1}^{n}x_{c}^{i}$$

• **Std:** the standard deviation of the two-dimension features from all trials of the EEG signals within a session.

(11) $$s=\sqrt{\frac{1}{2\,n}\,\sum_{c=1}^{2}\sum_{i=1}^{n}{\left|x_{c}^{i}-\mu \right|}^{2}}$$

• ***Dist_w_***: The within-class distance is the mean distance for all trials to the center of the corresponding class.

(12) $$D\,i\,s\,t_{w}=\frac{1}{2\,n}\,\sum_{c=1}^{2}\sum_{i=1}^{n}{||x_{c}^{i}-\mu_{c}||}_{2}$$

• ***Dist_b_***: The between-class distance is the distance between the center of the two classes.

(13) $$D\,i\,s\,t_{b}={||\mu_{1}-\mu_{2}||}_{2}$$

in which, μ_*c*_ is the mean value of the trials from class *c*, the operation of || returns the absolute value for each element in a vector, and || ||_2_ is the 2-norm of a vector. Since the feature dimension is 2 in this work, the statistics μ,*s* ∈ *R*^2^ and *Dist*_*w*_, *Dist*_*b*_ ∈ *R*. Firstly, the two-sample *t*-test was also performed on the four types of statistics from the diagonal elements between the 8 8 matrix in Exp1 and the two 10 10 matrices in Exp2. Both are the result of the within-session self-test. Secondly, the cross-subject result (non-diagonal elements in the 8 8 matrix in Exp1) were compared with the cross-session result (the nondiagonal elements in the two 10 10 matrices in Exp2). To avoid multiple comparison errors, Bonferroni correction has been applied with the new critical threshold $\alpha =\frac{0.05}{12}=4.17\times 10^{-3}$.

### 2.6. Classification-level analysis

In this part, based on the different machine learning assumptions, six types of training dataset selection strategies have been proposed, and the performances between the cross-subject and cross-session tasks were compared.

1) **Prev:** the previous session of the EEG signal was used for training. Suppose the state of the brain changes slowly. The signal from the previous session should be the most similar to the current EEG data. Hence, it would be suitable for training.
2) **Next:** the next session of the EEG signal was used for training. Similar to the Prev strategy, the EEG signal from the following session would also be similar to the current EEG data. While the Prev strategy is causal and available for online applications, the Next strategy is noncausal and cannot be applied online. It should be noted that both the Prev and Next strategies were only applied to the cross-session tasks.
3) **Best:** the session with the best self-test accuracies was used for training. The Best strategy that supposes the data with the highest self-test accuracy would be to make the optimized spatial filter and classifier.
4) **Worst:** the session with the worst self-testing accuracies was used for training. Different from the Best strategy, the Worst strategy supposes that more difficulty during the training would make the spatial filter and classification more powerful for the cross-subject and cross-session generalization.
5) **Closest:** the session with the smallest Kullback–Leibler Divergence (KL Divergence) with the testing set was used for training. The Closest strategy supposes more similarities between the training and testing dataset leads to better classification accuracy.
6) **All:** all the sessions except the testing session were used for training. The All strategy supposes a larger sample size from the training set would lead to better classification accuracy.

## 3. Results

### 3.1. Experiment performance

In the online experiment with the paradigm in Figure 2, the first four runs were used for training, and the last four runs were used for testing for each session of the experiment. CSP was used for feature extraction and LDA was used as the classifier. The feature dimension, also known as the number of spatial filters, was set to six for the online experiment. To facilitate the comparison with the performance of subsequent cross-subject, and cross-session tests, the feature dimension was reduced from 6 to 2 and the whole online schema has been run again offline.

**FIGURE 3:**
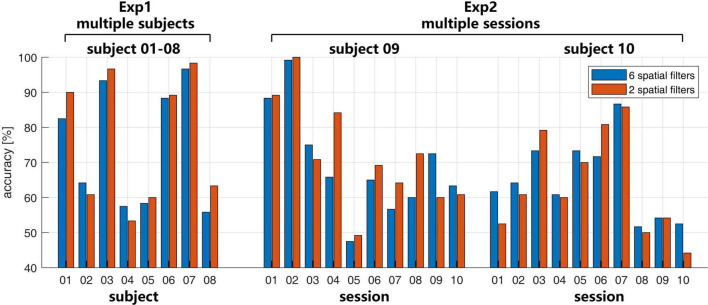
The recognition accuracies with the number of spatial filters 6 (blue bar) and 2 (red bar) in Exp1 and Exp2.

In Exp1 in Figure 3, the recognition accuracy was 74.58 ± 17.34% with 6 spatial filters and 76.45 ± 18.72% with 2 spatial filters. The paired two-sample *t*-test shows no significant difference between the two conditions (*p* = 0.26). Large inter-subject variability of the BCI performance has been shown, in which the accuracies on subjects 01, 03, 06, and 07 were higher than 80%, but the accuracies on subjects 04, and 05 were lower than 60%.

In Exp2 in Figure 3, the large intra-subject variability also happened on both subjects 09 and 10. Both two subjects have several sessions with accuracies higher than 80% or lower than 60%. For subject 09, the mean accuracies were 69.33 ± 15.21% with the 6 spatial filters and 72.00 ± 15.23% with the 2 spatial filters. For subject 10, the mean accuracies were 65.00 ± 18.72% with the 6 spatial filters and 63.75 ± 14.41% with the 2 spatial filters. Similar to the case in the multi-subject test in Exp1, there is no significant difference for the different number of spatial filters with *p* = 0.36 for subject 09 and *p* = 0.50 for subject 10.

### 3.2. Signal-level results

**FIGURE 4:**
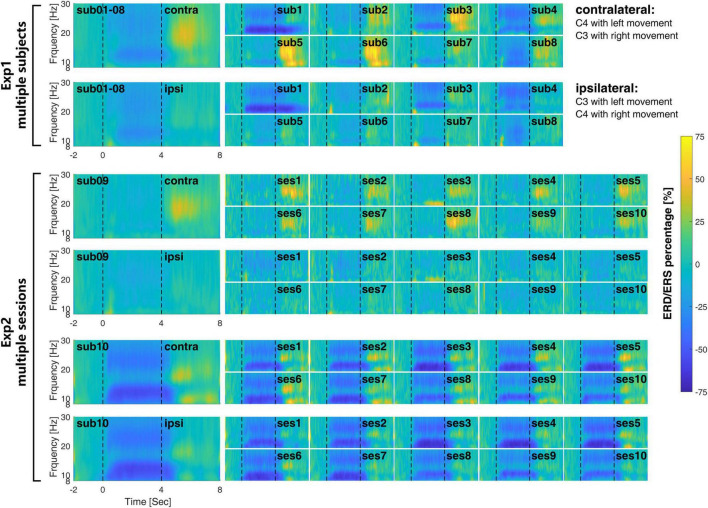
The inter- and intra-subject variability of the time-frequency response during the MI-BCI. The large figures on the left are the averaged time frequency response cross-subject in Exp1 and cross-session in Exp2. The inter-subject variability in Exp1 is much larger than the intra-subject variability in Exp2.

**FIGURE 5:**
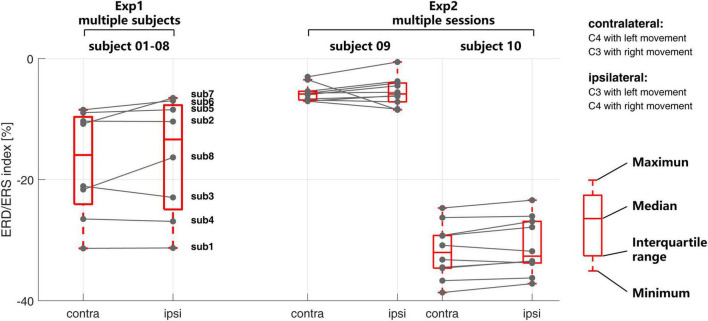
The inter- and intra-subject variability of the event-related desynchronization/synchronization (ERD/ERS) index in Exp1 and Exp2.

At the signal level, Figure 4 shows the time-frequency response for each session of the EEG signal in both multi-subject experiment (Exp1) and multi-session experiment (Exp2). The corresponding ERD/ERS indexes are shown in Figure 5.

At the subject level, the time-frequency response differs greatly from each other. For subject 01, μrhythm ERD was observed at both the contralateral and ipsilateral side and remained 2 or 3 s after the end of motor imagery. Most of the subjects show clear β rhythm ERS on the contralateral side after the finishing of motor imagery, which is clearer on subjects 03 and 05. It is interesting to find that on the contralateral side of subjects 01 and 04, there is μrhythm ERD and β rhythm ERS appear at the same time after the finishing of motor imagery.

At the session level, the time-frequency response from different sessions within subjects 09 and 10 keep more consistent from each other than Exp1. The ERD/ERS index in Figure 5 also shows the same situation. As compared with the time-frequency response from different subjects in Exp1, the time-frequency response in different sessions in Exp2 shares a similar time and frequency scale within the subject. The ERD during the time of motor imagery from all sessions on subject 09 is weaker than subject 10 at both the contralateral and ipsilateral sides.

Compared with the recognition accuracies in Figure 3, in Exp1, a larger ERD/ERS may not necessarily correspond to a higher recognition rate, such as subject 04. In Exp2, the consistent time-frequency response also may not correspond to a similar recognition rate.

### 3.3. Feature-level results

**FIGURE 6:**
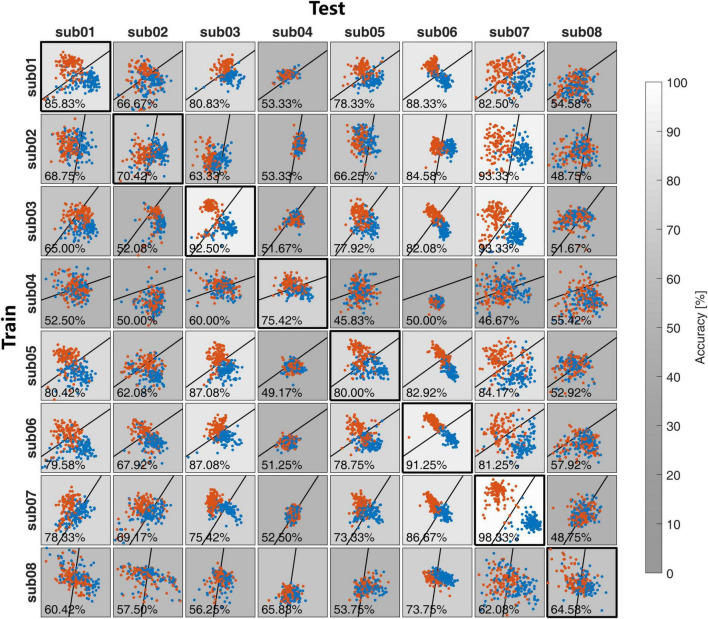
The feature distribution and the classification accuracies in the cross-subject in Exp1.

**FIGURE 7:**
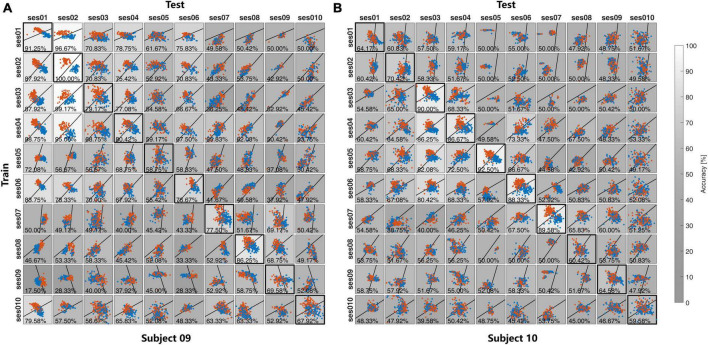
The feature distribution and the classification accuracies in the cross-session in **(A)** Subject 09 and **(B)** Subject 10 in Exp2.

In both Exp1 and Exp2, each session of the EEG data was used to make the spatial filters and classifier and applied to the EEG data from other subjects in Exp1 and other sessions of the same subject in Exp2. Both the feature distributions and the recognition accuracies were illustrated in Figure 6 for Exp1, and Figure 7 for Exp2. The grayscales indicate the recognition accuracies. To show the difference in feature distribution between Exp1 and Exp2, the statistical mean, Std, within-class distance, and between-class distance were compared. The diagonal and non-diagonal elements in Figures 6, 7 were compared separately for the self-test and cross-subject/session test in Table 1.

**TABLE 1:** *t*-test result on the statistical of Mean, Std, *Dist_w_*, *Dist_b_* for CSP features.

Task	Statistical		Exp1 multiple-subject	Exp2 multiple-session	*t*-value	*p*-value
self-test	Mean	dim1	-2.29	-2.22	-1.70	0.10
		dim2	-2.33	-2.31	-0.29	0.77
	Std	dim1	0.68	0.55	2.05	0.05
		dim2	0.65	0.57	1.37	0.18
	*Dist*_*w*_		0.57	0.56	0.25	0.80
	*Dist*_*b*_		1.20	0.81	1.71	0.10
Cross subject/session test	Mean	dim1	-2.40	-2.55	2.33	0.02
		dim2	-2.49	-2.58	1.14	0.26
	Std	dim1	0.51	0.41	**4**.**45**	**1.34** × **10**^–5^
		dim2	0.50	0.42	**3**.**42**	**7.26** × **10**^–4^
	*Dist*_*w*_		0.54	0.47	2.94	3.60 × 10^–3^
	*Dist*_*b*_		0.52	0.29	**4**.**89**	**1.84** × **10**^–6^

*Bonferroni correction is applied for multiple comparison errors. The Bonferroni-adjusted alpha was set to be 0.05/12=4.17×10^−3^. Bold values denote statistical significance at the *p* < 0.05 level.

For the self-test result, as is shown in Table 1, there is no significant difference for all between multiple-subject and multiple-session experiments. All these statistics reflect the feature distribution of the diagonal elements in Figures 6, 7. Except for different subjects, these diagonal elements are all the results of within-session tests without any difference.

For the cross-subject and cross-session test, there is no significant difference in the Mean value between the cross-subject test in Exp1 and the cross-session test in Exp2, but the *p*-value of 1.34×10^−5^ and 7.26×10^−4^ indicate the difference of the std value on both two feature dimensions. As compared with the self-test result, the Std value from both Exp1 and Exp2 was reduced in the cross-subject/session test. Further exploration indicated that the reduction of Std mainly contributed to the reduction of *Dist_b_*. As compared with the result in the self-test, in the cross-subject/session test the value *Dist_b_* decreased from 1.19 to 0.52 for Exp1 and 0.81–0.29 for Exp2. While the reduction of *Dist_w_* value was not so great and also the difference of *Dist_w_* value between the cross-subject test in Exp1 and the cross-session test in Exp2 was not significant with the *p*-value of 3.60×10^−3^, which is higher than the Bonferroni-adjusted alpha threshold $\alpha =\frac{0.05}{12}=4.17\times 10^{-3}$.

### 3.4. Classification-level results

In this part, we did not compare the performance between the different techniques of transfer learning but focused on investigating the difference between the cross-subject test in Exp1 and the cross-session test on the classifier level. Hence, the simplest LDA classifier was applied for ease to understand, and the effect of training set selection strategies was compared to the difference between the cross-subject test in Exp1 and the cross-session test in Exp2.

As is shown in Table 2 for the cross-subject test in Exp1, the Best strategy shows the best recognition accuracies of 72.93% on average, which is much better than 68.28% from the Closest strategy. The All strategy also achieves a pretty good performance of 72.55%. The Worst strategy is the worst.

**TABLE 2:** The performance of the different strategies in the cross-subject test in Exp1.

Subject	Recognition accuracies (%)			
	Closest	Best	Worst	All
sub01	80.42	79.33	52.50	82.92
sub02	62.08	69.17	50.00	65.42
sub03	56.25	75.42	60.00	79.17
sub04	49.17	52.50	75.42	48.75
sub05	78.33	73.33	45.83	75.00
sub06	77.92	86.67	50.00	89.58
sub07	93.33	98.33	46.67	86.67
sub08	48.75	48.75	55.42	52.92
Mean	68.28	**72.93**	54.48	72.55

Bold values denote the maximum accuracy.

The situation in the cross-session test in Exp2 would be different. As is shown in Table 3, the All strategy achieves the best results on both subjects 09 and 10. The performance of the strategies Prev, Next, Closest and Best are similar, which was lower than the All strategy by 4–12%. Again, the Worst strategy is the worst.

**TABLE 3:** The performance of the different strategies in the cross-session test in Exp2.

Session	Recognition accuracies (sub09/sub10)					
	Prev	Next	Closest	Best	Worst	All
ses01	None	87.92/60.42	87.92/48.33	87.92/54.58	72.08/48.33	81.67/59.17
ses02	96.67/60.83	99.17/65.00	95.00/51.67	100.0/38.75	56.67/47.92	99.17/70.83
ses03	70.83/58.33	68.75/86.25	56.67/86.25	70.83/40.00	56.67/39.58	80.00/81.67
ses04	77.08/68.33	68.75/72.50	67.92/51.67	75.42/46.25	68.75/50.42	77.92/61.25
ses05	59.17/49.58	55.42/57.92	55.42/57.92	52.92/50.42	58.75/48.75	58.33/71.25
ses06	58.33/66.67	43.33/67.50	66.67/45.42	70.83/67.50	58.33/45.42	73.33/65.42
ses07	41.67/52.92	52.92/50.00	63.33/50.42	43.33/89.58	47.50/53.75	75.00/72.92
ses08	51.67/65.83	58.75/51.67	63.33/50.00	53.75/65.83	48.33/45.00	78.75/68.33
ses09	68.75/53.75	52.92/46.37	37.08/53.75	42.92/60.00	37.08/46.67	51.25/62.50
ses10	52.08/47.92	None	50.42/47.92	50.00/51.25	50.42/59.58	53.33/54.58
Mean	64.02/58.24	65.32/61.95	64.37/54.33	64.79/56.41	55.45/48.54	**72.87/66.79**

Bold values denote the maximum accuracy.

## 4. Conclusion and discussion

Inter- and intra-subject variability played an important role in the EEG signal decoding for the BCI application, which is also the current research frontier. Several methods, especially deep learning methods, have been proposed to fix inter- and intra-subject variability for transfer learning. However, most work has noticed the difference existing in the feature distribution between the training samples and test samples. But to the best of our knowledge, there has been no work to investigate the differences between cross-subject and cross-session tests. In this work, we investigated the problem of inter- and intra-subject variability at different levels.

Firstly, with the construction of the online experiment platform, both multi-subject and multi-session experiment has been conducted. The result shows the large variability of the online classification in both multi-subject (Exp1) and multi-session (Exp2) experiments. The result of the large inter-subject variability agrees with existing studies of BCI inefficiency (Zhang et al., 2020), that part of the subject is unable to make their brain signals decoded by the current BCI algorithm. But the larger intra-subject variability has not been fully investigated in the existing work. Compared with the high cross-session reliability recognition accuracies from the 93 subjects 2 sessions experiment in the previous work (Liu et al., 2020), the high intra-subject variability in this work may be attributed to the subjects’ adaptation process of motor imagery.

Secondly, the signal level investigation on the inter- and intra-subject variability shows the high variability of the time-frequency response among different subjects in Exp1 for both the time-frequency scale and amplitude of the response. However, the multi-session result in Exp2 shows the consistency of time-frequency response among different sessions within the subject. The ERD/ERS index also indicated the same conclusion. Further, it is found that the time-frequency response may not be directly related to the classification accuracies. In the multi-subject test in Exp1, both the lower ERD/ERS index and the larger difference between the contralateral and ipsilateral sides are not correlated to the recognition accuracies. Some subjects could induce large ERD/ERS signals but still have poor BCI recognition results. This result proved that the BCI inefficiency, or so-called BCI illiteracy, was not due to the inability of the subject to regulate their own brain signal during the motor imagery, but the inability of the current algorithm to decode the EEG single efficiently.

Thirdly, the feature level and classification level investigation reveal the different characteristics during the transfer learning between cross-subject and cross-session tests. With the CSP spatial filtering, the Std values from both two dimensions are significantly different between the cross-subject test in Exp1 and the cross-session test in Exp2, which is mainly caused by the reaction of the between-class distance *Dist_b_* in the cross-session test. With the LDA classifier, it is found that the quality of the training sample with the Best strategy is important for the cross-subject, which could be attributed to the larger inter-subject variability. While the situation in the cross-session test is different, with the consistent time-frequency response cross-session, the larger sample size in the All strategy would be more helpful to improve the cross-session recognition accuracies.

### 4.1. Limitation

In this work, we investigated the cross-session and cross-subject variability of motor imagery BCI. However, the problem of cross-run variability has not been discussed. Cross-run variability, as known as within-session variability, is also key important to the BCI application. It is found that the cross-run variability would make the performance of the BCI decoding algorithm decrease. We have tried an unsupervised adaptation algorithm with a fuzzy C-means method to catch up with the cross-run variability in our previous study (Liu et al., 2012). However, the limited sample size of 30 trials each run (15 trials per class) prevents us from performing the same study on cross-run variability as we did on cross-session and cross-subject variability.

## Data availability statement

The raw data supporting the conclusions of this article will be made available by the authors, without undue reservation.

## Ethics statement

The studies involving human participants were reviewed and approved by Medical Ethics Committee, Health Science Center, Shenzhen University. The patients/participants provided their written informed consent to participate in this study.

## Author contributions

All authors listed have made a substantial, direct, and intellectual contribution to the work, and approved it for publication.
